# Supplementary material for: Effects of two-week e-learning on eHealth literacy: a randomized controlled trial of Japanese Internet users
Source: PeerJ. 2018 Jul 13;6:e5251. doi: 10.7717/peerj.5251 (PMC6047505; doi:10.7717/peerj.5251)
Supplement: Supplemental Information 2 [file peerj-06-5251-s003.docx]

| Dependent variable | Value | Intervention group (*n* = 110) | Control group (*n* = 148) | Intervention v.  Control Δ change^a^ and Cohen’s d (95% Confidence Interval) *p*-value |
| --- | --- | --- | --- | --- |
| The eHealth Literacy Scale, eHEALS (mean (SD)) | Baseline | 24.5 (6.59) | 25.9 (6.18) | 1.81 (0.24, 3.38) 0.286 (0.04, 0.53) *p* = 0.024 |
|  | Follow-up | 27.0 (5.92) | 26.6 (5.63) |  |
|  | Score change | 2.55 (7.56) | 0.74 (5.25) |  |
| The Healthy Eating Literacy Scale, HEL (mean (SD)) | Baseline | 3.41 (0.70) | 3.52 (0.70) | -0.03 (-0.19, 0.12) -0.05 (-0.30, 0.19) *p* = 0.680 |
|  | Follow-up | 3.52 (0.59) | 3.65 (0.54) |  |
|  | Score change | 0.11 (0.67) | 0.14 (0.59) |  |

Table.S1 Means, standard deviations (SD), change scores (follow-up minus baseline), and

intervention effects (Δ change)^a^ compared to control group by per protocol analysis.

^a^ Score change of intervention group minus score change of control group

Table.S2 Results on evaluation skill at baseline and follow-up, intervention effect, and

comparison of intervention group to control group by per protocol analysis.

| Variable | Value | Intervention group (*n* = 110) | Control group (*n* = 148) | Intervention v. Control Relative Risk Ratio (95% Confidence Interval)  *p*-value |
| --- | --- | --- | --- | --- |
| Having evaluation skill  (n (%)) | Baseline | 35 (31.8) | 47 (31.8) |  |
|  | Follow-up | 64 (58.2) | 46 (31.1) |  |
| Change in evaluation skill  (n (%)) | Better | 36 (32.7) | 19 (12.8) | 3.08 (1.64, 5.81) *p* < 0.001 |
|  | No change | 67 (60.9) | 109 (73.6) | (Reference outcome) |
|  | Worse | 7 (6.4) | 20 (13.5) | 0.57 (0.23, 1.42) *p* = 0.227 |
