# Supplementary material for: Effects of two-week e-learning on eHealth literacy: a randomized controlled trial of Japanese Internet users
Source: PeerJ. 2018 Jul 13;6:e5251. doi: 10.7717/peerj.5251 (PMC6047505; doi:10.7717/peerj.5251)
Supplement: Supplemental Information 3 — Manuscript of e-learning contents [file peerj-06-5251-s004.pdf]

e-Learning Contents for eHealth Literacy.

Note: This document is a draft for the preparation of e-learning contents. Revisions may be made to its text and layout when it is marked up for HTML. In addition, the embedded images of Japanese search results and bibliographic information will remain in Japanese in the English version.

Content

1. Unreliable Information on the Web 3

1.1. Introduction 3

1.2. The Revenue Sources of Suspect Health Information Websites 4

1.3. How to Evaluate Articles about Health Information on the Internet 4

Provocative content increases page views 4

Who authored this health information? 5

How to evaluate health-related articles individually 5

1.4. Points to Remember for Internet Searches 5

Search keywords you should add 5

What's the quality of the site I found? 6

How can I search for papers by myself? 6

1.5. Study Questions 7

2. The Difference Between Anecdote and Science 9

2.1. Introduction 9

2.2. You Can't Count on Anecdotes 9

You won't find negative testimonials 9

Anecdotal evidence is unreliable 9

2.3. You Can Count on Scientific Research 10

Check for a research report 10

2.4. Introduction of Research Design 11

Randomized controlled trial 11

Cohort study 12

Case report 13

2.6. Study Questions 13

3. Words of Caution about Sharing Information on Social Media 14

3.1. Introduction 14

3.2. Exercise Caution When Sharing Information 14

Don't be too quick to share and spread information 14

3.3. Echo Chambers 14
What is an "echo chamber"? 14
Echo chamber formation process 14
The harmful effects of echo chambers 15
3.4. Study Questions 15
4. Cognitive Biases and Points to Remember When Dealing with Health Information 17
4.1. Introduction 17
4.2. Five Kinds of Cognitive Biases 17
1. Seeing patterns where there are none 17
2. Seeing causal relationships where there are none 17
3. Focusing on information that fits the hypothesis 17
4. Being influenced by preconceptions 17
5. Focusing on easy-to-remember information 18
4.3. Points to Remember When Dealing with Health Information 18
1. Doubt by default 18
2. Be wary of typical language used by inaccurate claims and low-quality websites 18
3. Be wary of the identity of the website owner 18
4. Make sure you're not reading marketing for fake health products 19
5. Regard claims only supported by testimonials as lacking scientific value 19
6. Check for published scientific research regarding health claims 19
7. Investigate the content of other websites as well 19
8. Remember your own vulnerability to cognitive biases 19
4.4. Study Questions 19
4. Summary 21
Book Guide (Japanese Books) 21
Book Guide (English Books) 21

1. Unreliable Information on the Web

1.1. Introduction

When you search for health-related information on the Internet, some untrustworthy websites show up at the top of the search results. Why do search engines rank such sites prominently, even though they contain unreliable information?

Let's take a look at a real-world example for the search term "milk". (Google search results on September 3, 2017)

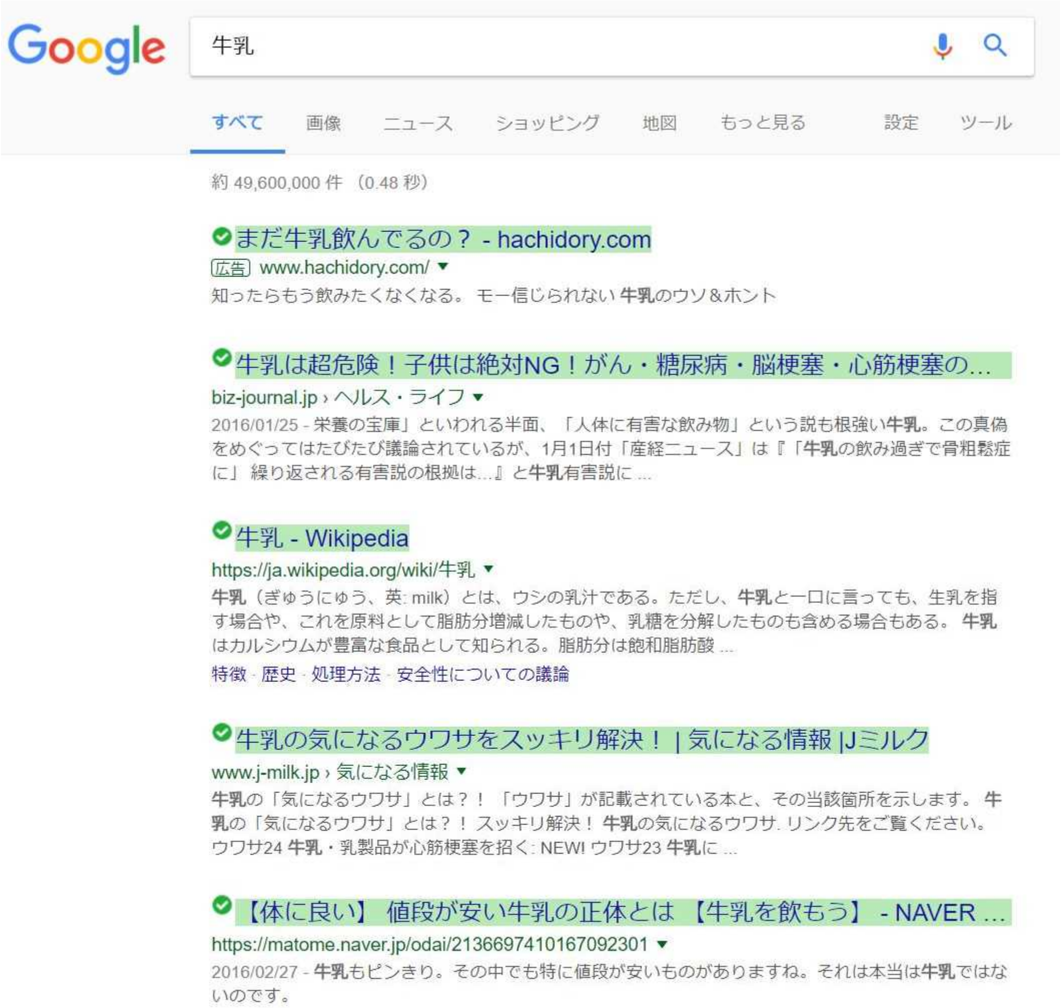

The top two hits are negative in tone. The first result, an advertisement, reads: "Still drinking milk? You won't want to anymore once you hear this." The second result claims milk is harmful to your health: "Milk is super dangerous! Never let your children drink it!" However, research has generally shown milk to be good for your health. Why do articles that haven't taken into account such research findings appear near the top of the search engine results?

For emphasis, here is a selection of studies that report the beneficial health effects of milk.

1. Umesawa et al. Dietary calcium intake and risks of stroke, its subtypes, and coronary heart disease in Japanese: the JPHC Study Cohort I. *Stroke*. 2008 Sep;39(9):2449-56.
2. Liu S et al. Dietary calcium, vitamin D, and the prevalence of metabolic syndrome in middle-aged and older U.S. women. *Diabetes Care*. 2005 Dec;28(12):2926-32.
3. Kazuhiro U, et al. Milk, Dairy Products and Metabolic Syndrome: A Cross-sectional Study of Japanese. *J Jpn Soc Nutr Food Sci*. Vol. 63 (2010) No. 4. 151-159.

1.2. The Revenue Sources of Suspect Health Information Websites

Why do unreliable articles appear near the top of search engine results? To understand, we need to first ask the question, "How do these sites generate income?"

- *Revenue from product sales (promoting fake health products)*
  Owners of some websites make money by selling products or goods. These kinds of predatory sites typically sell expensive health-related products that lack medical efficacy: so-called *fake health products*. They rarely provide scientific evidence (i.e., research publications) for their claims.
- *Affiliate revenue*
  Articles on some websites contain links that send the user to an Internet-based shop. This system is designed to encourage visitors to make purchases at the linked shop. When the user buys a product, the site owner receives a portion of the proceeds.
- *Advertising revenue based on page views*
  Website owners can receive revenue when a visitor views or clicks advertisements located in articles hosted on their own site, proportional to the number of clicks/views.

Writing high-quality and medically-accurate articles is not a guarantee that a website owner will make money from any of these revenue models. However, website operators have many techniques at their disposal to ensure their content gets ranked high among search results. Their ability to make money with misleading and fraudulent information is why even unreliable articles can appear at the top of search results.

1.3. How to Evaluate Articles about Health Information on the Internet

Provocative content increases page views

Website owners who host fraudulent health information seem to know that the more radical their content, the more page views they get. This tendency incentivizes them to publish provocative articles and information to try to get their website ranked higher among search results. The reason so many untrustworthy articles are ranked at the top of the search results is the abundance of site operators who try to increase their page views and obtain revenue from advertising fees.

Who authored this health information?

When considering the reliability of information you encounter on the Internet, you also need to ask yourself, “Who wrote this?” Articles on low-quality websites are often unattributed: the author is unknown. One health information website experienced a scandal in 2016 when it was discovered that the articles it hosted were largely written via crowdsourcing, not by professionals. Make sure to check who wrote the articles you encounter on the Internet: confirming the author’s name and position is good practice.

How to evaluate health-related articles individually

Is the article logically consistent?

Poor-quality articles can exhibit inconsistent logic and flow, because they are often prepared by assembling sentences and paragraphs from many different sources. When an article’s logic is inconsistent, you should assume you can’t trust the health information presented.

Does the article have objective data?

Whether the health information provided by an article is based on objective data is an important consideration when evaluating its trustworthiness. You can interpret the information in an article to lack objectivity, and therefore trustworthiness, in the following cases.

- The article relies heavily on subjective information such as personal impressions.
- The article presents an individual’s beliefs, in the absence of any research evidence to substantiate it.
- The article does not provide any objective reference material.
- The article’s topic has been researched, but its interpretation of the findings is distorted.

Check whether a claim has been researched

It is difficult to determine whether or not health claims or products have been researched. When you encounter suspect health information, the best approach is to search again, and try to corroborate those claims or findings based on the content of other websites.

1.4. Points to Remember for Internet Searches

Search keywords you should add

Restrict which sites you search

You can change your search conditions to only include site domains ending in “ac.jp,” “go.jp,” and “lg.jp.” Domains ending in “ac.jp” indicate a site operated by an educational institution (such as

universities); “go.jp,” is a site oerated by a government institution (such as the Ministry of Health, Labour and Welfare); and “lg.jp,” by local public entities (such as public health centers). These kinds of organizations have no incentive to create false or misleading health information; rather, they tend to try to publish information that is as accurate as possible. Adding such search conditions allows you to filter out incorrect information from your results.

Search for opposing opinions

If your search terms lead you to health information that seems suspicious, you can add negative keywords such as “fake” or “fraudulent” to your original search. This should bring any criticisms of the suspect health information into your search results. You can review the trustworthiness of the original claims by checking them against health information found with these conditions.

What’s the quality of the site I found?

How to determine a site’s quality

No matter the type, high-quality information is always accompanied by evidence. You can judge health and medical information to be high quality if it is accompanied by scientific evidence. You can judge citations—references of investigations that yielded the information—as positive proof that a claim has evidence.

Is there a citation?

Health information should have citations if it is backed by scientific research, published in the form of conference reports or scholarly papers. Perhaps you aren’t familiar with citations: take a look below to see what one looks like. It’s important that you check whether citations accompany health information on the Web.
Example: Harada A et al. Cost and effectiveness of exercise therapy for patients with essential hypertension. Japanese Journal of Public Health, 48(9), 753-763.

How can I search for papers by myself?

When you can’t find a citation

Sometimes you might be unable to find citations in a given article. In these situations, you might want to search for the scientific evidence yourself. This is a difficult task, but it’s simpler now than ever before.

Sites you can use for free

Google Scholar is one website you can use to search for reference works for free. You can search

for research findings over a wide range of research papers and other documents that can serve as scientific evidence.

Let’s look at the results for “exercise” and “hypertension” in Google Scholar. Based on the search results, you can learn what kind of research is available on the topic in question. However, there are weaknesses to this approach: sometimes you can’t actually see the detailed content of the studies shown, and even if you can, it may be difficult to evaluate the strength of the scientific evidence presented.

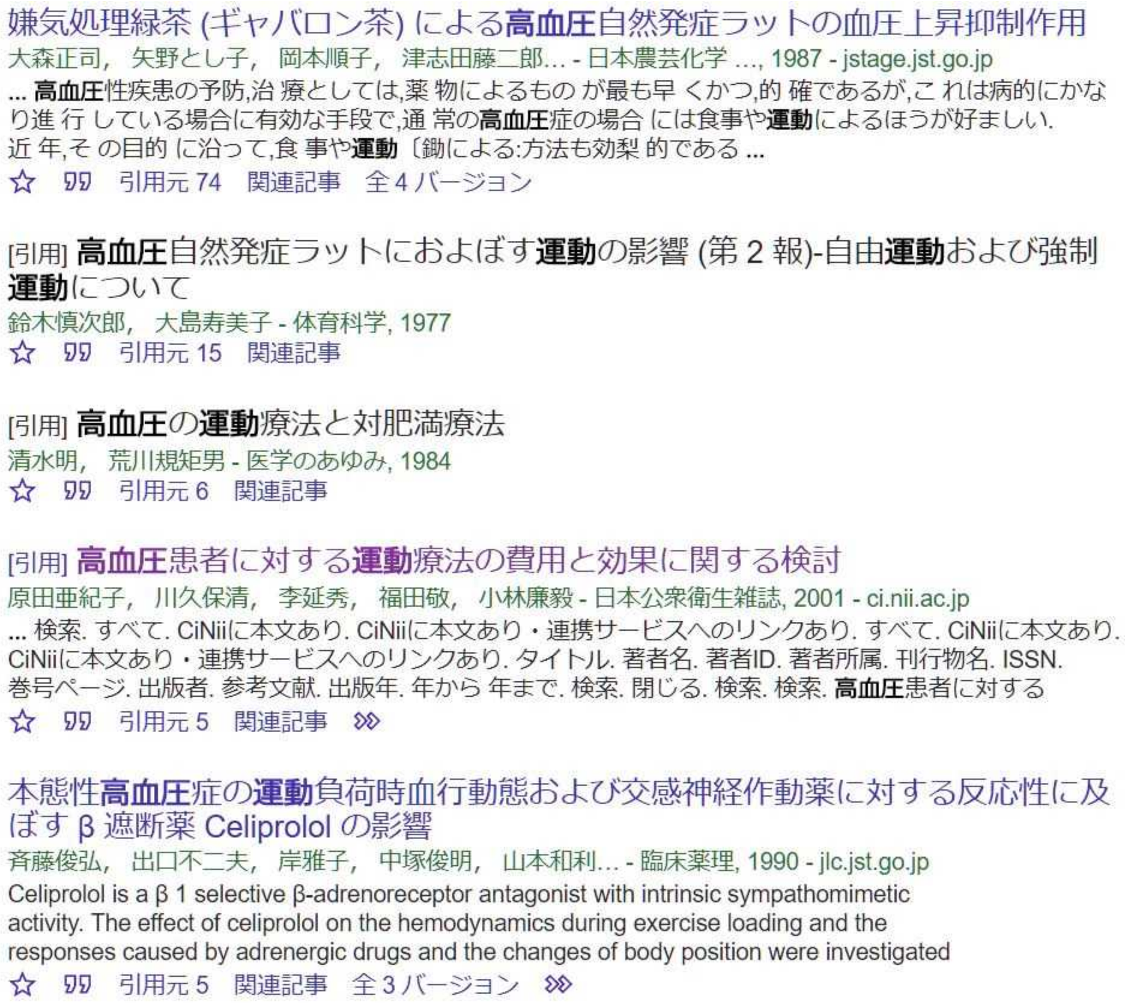
嫌気処理緑茶 (ギャバロン茶) による**高血圧**自然発症ラットの血圧上昇抑制作用
大森正司， 矢野とし子， 岡本順子， 津志田藤二郎… - 日本農芸化学 …, 1987 - jstage.jst.go.jp
… **高血圧**性疾患の予防,治 療としては,薬 物によるもの が最も早 くかつ,的 確であるが,こ れは病的にかなり進 行 している場合に有効な手段で,通 常の**高血圧**症の場合 には食事や**運動**によるほうが好ましい.
近 年,そ の目的 に沿って,食 事や**運動**〔鍬による:方法も効梨 的である …
☆ 99 引用元 74 関連記事 全 4 バージョン

[引用] **高血圧**自然発症ラットにおよぼす**運動**の影響 (第 2 報)-自由**運動**および強制**運動**について
鈴木慎次郎， 大島寿美子 - 体育科学, 1977
☆ 99 引用元 15 関連記事

[引用] **高血圧**の**運動**療法と対肥満療法
清水明， 荒川規矩男 - 医学のあゆみ, 1984
☆ 99 引用元 6 関連記事

[引用] **高血圧**患者に対する**運動**療法の費用と効果に関する検討
原田亜紀子， 川久保清， 李延秀， 福田敬， 小林廉毅 - 日本公衆衛生雑誌, 2001 - ci.nii.ac.jp
… 検索. すべて. CiNiiに本文あり. CiNiiに本文あり・連携サービスへのリンクあり. すべて. CiNiiに本文あり. CiNiiに本文あり・連携サービスへのリンクあり. タイトル. 著者名. 著者ID. 著者所属. 刊行物名. ISSN. 巻号ページ. 出版者. 参考文献. 出版年. 年から 年まで. 検索. 閉じる. 検索. 検索. **高血圧**患者に対する
☆ 99 引用元 5 関連記事 »

本態性**高血圧**症の**運動**負荷時血行動態および交感神経作動薬に対する反応性に及ぼす β 遮断薬 Celiprolol の影響
斉藤俊弘， 出口不二夫， 岸雅子， 中塚俊明， 山本和利… - 臨床薬理, 1990 - jlc.jst.go.jp
Celiprolol is a β 1 selective β-adrenoreceptor antagonist with intrinsic sympathomimetic activity. The effect of celiprolol on the hemodynamics during exercise loading and the responses caused by adrenergic drugs and the changes of body position were investigated
☆ 99 引用元 5 関連記事 全 3 バージョン »

1.5. Study Questions

Please select **all of the choices** below that demonstrate **incorrect** assumptions about health information on the Internet. (*Choices are displayed in random order)

- Websites containing high-quality health information have high numbers of visitors (page views).
  - [Correct answer] Commentary: High-quality health information does not necessarily result in greater traffic to (more page views of) websites.

- I found a health-related article by performing a Web search. However, it was unclear who wrote it, so its content probably isn’t reliable.
  - Commentary: You can’t really trust health-related articles when you don’t know the identity or position of the author.
- The supplement I found has many testimonials from real users of its website, so I thought the information written about it could be trusted.
  - [Correct answer] Commentary: No matter how numerous, user testimonials and anecdotes don’t mean claims about a health product can be trusted.
- My Web search resulted in too many hits, and I didn’t know which sites I could trust. So, I added “go.jp” and “ac.jp” to my search keywords to narrow down the results.
  - Commentary: You can refine search engine criteria to include only university and government sites, ensuring that only sites with a low likelihood of fabrication (i.e., high trustworthiness) will appear in the results.

2. The Difference Between Anecdote and Science

2.1. Introduction

There is a lot of false and misleading health information on the Internet. This broadly covers claims exhorting the therapeutic or health-promoting effects of a product or treatment, despite a lack of accurate evidence or scientific support. Under ordinary circumstances a drug, product, or service's therapeutic efficacy or health-promoting effects should be based strictly on scientific evidence. However, the makers of fraudulent claims don't adhere to this process: they publish testimonials, mere opinions, and similarly biased information on the Internet to convince consumers of the efficacy of their product.

This section introduces the differences between anecdotes and science.

2.2. You Can't Count on Anecdotes

You won't find negative testimonials

Websites that provide information about supplements or health tips typically publish only positive anecdotes and customer impressions, not negative ones. The goal of these positive testimonials is marketing. Supplement sellers don't publish the negative testimonials or complaints they receive from customers, they just delete them, picking and choosing only the positive ones to post. Screening out negative reviews can yield an abundance of good anecdotes supporting a supplement or treatment's efficacy, but they can't be relied upon.

Anecdotal evidence is unreliable

Just because a claim is accompanied by a lot of anecdotes doesn't make it any more trustworthy. Let's go through a few reasons why a collection of personal testimonials is not evidence that the product or service is reliable or effective.

1. **Maybe the user's health would have improved without doing anything.**
   Nobody knows if the health food or supplement is really responsible for the user's improved health. Perhaps they would have gotten better on their own, without it.
2. **It's not certain that the user was really sick.**
   In some cases, it's difficult to determine whether the user really had the disease they claim. One example is when a doctor diagnoses a patient with "suspected cancer." This medical term just means that a finding *might be* cancerous. However, a frightened patient is liable to feel they were just definitively diagnosed with cancer. We can imagine such a patient making the following inferences based on their misunderstanding.
   ① "I was diagnosed with cancer." (Reality: The doctor said, "You might have cancer.")
   ② "I used the supplement."

③ "My cancer got better." (Reality: It was never cancer in the first place.)

3. **The user assumes the supplement healed their cancer.**

   Their cancer may have been cured by a standard therapy, performed at the same time they were taking the supplement. Users might not mention in their testimonials that they also received another, standard treatment for their disease. In other cases, users might attribute their recovery to a health food or supplement, even though they were receiving a standard treatment (e.g., anti-tumor drugs) at the same time.

4. **Their disease might not really be cured.**

   In some cases, a user's disease might not really have been cured, despite their testimonial proclaiming otherwise. Their testimonial might only include their story up to the point where they felt they "got better," but not the part where their condition deteriorated afterwards. For example, let's consider the following sequence of events. The user might have written their positive testimonial after the third step. If they had written it later, after the sixth step, the testimonial probably would have concluded that the supplement was not responsible for their recovery.

   ① Disease detected.
   ② Supplement taken.
   ③ Health improves.
   ④ Health worsens.
   ⑤ Standard treatment (e.g., surgery)
   ⑥ Recovery.

2.3. You Can Count on Scientific Research

Unlike anecdotes, scientific research evaluates both positive and negative findings observed for a given treatment approach. Drugs, health products, and treatments need to be examined by scientific means to determine whether or not they are beneficial to health. Such methods don't require the researchers to uncover the physiological mechanisms responsible for those effects, or determine the reason why a treatment works. Scientific techniques dispense with preconceptions, like assuming a drug is good for your health. Such procedures simply evaluate two things in an objective manner: the treatment approach, and whether or not its recipients get better.

Check for a research report

Make sure you check whether the health information you find is associated with a research report that backs up its claims with scientific evidence. You can review how to search for research reports and other scholarly works in the section "Points to Remember for Internet Searches" in the module "Unreliable Information on the Web" which includes instructions on how to search for these kinds of reports.

2.4. Introduction of Research Design

This section introduces typical research designs used in medical research. Many exist; we will cover the following three:

1. Randomized controlled trial (RCT)
2. Cohort study
3. Case report

Randomized controlled trial

How to perform a randomized controlled trial

You can clarify the effects of a given treatment—for this example, let's say a drug—using the following process, called a *randomized controlled trial*. First you would need to gather a large sample of patients, and randomly assign them to two groups: those who take the drug, and those who take something similar (a dummy drug, or *placebo*). Next, you have these patients actually take the drug or placebo, and check whether or not their conditions improve. You could conclude that the drug is indeed effective if you observed a difference in therapeutic efficacy between patients who took the drug compared to those who took the placebo.

Notes and features of randomized controlled trials

That being said, people who take a drug or placebo can sometimes get better simply due to their belief that their treatment will be effective. This phenomenon is called the *placebo effect*. To avoid it, you need to make it so neither the patients receiving the treatment, nor the doctors administering it, know whether they are using the real drug or the placebo. This condition is known as *double blind*.

The randomized controlled trial is a superior research design because it can reveal for certain whether a given treatment works, even if the reason why it works or the responsible mechanism is unknown. For example, scurvy is cured if you eat lemons, even if you don't know anything about its active ingredient, vitamin C.

(Example) James Lind's discovery of a cure for scurvy

In 1746, James Lind conducted the world's first randomized controlled trial. At the time, England's Royal Navy was plagued by a disease called scurvy. Many sailors would come down with it and die while on voyages at sea. Now it is known that scurvy is caused by a lack of vitamin C, but it was strange and unknown at the time.

One day, Lind found that 12 sailors had come down with scurvy. Instead of giving all 12 men the same treatment, he divided them into six groups of two men, each receiving a different treatment.

① Apple cider

② Sulfuric acid solution (“elixir vitriol”)
③ Vinegar
④ Sea water
⑤ Medicinal (herbal) paste
⑥ Oranges and lemons

After a few days, the two men in the sixth group recovered. Lind thought, “I don’t know the reason why, but oranges and lemons are good for scurvy.” Vitamin C was the active ingredient in this cure, but the compound’s existence was still unknown in Lind’s era. This example shows how a randomized controlled trial can prove the efficacy of a treatment, even if the reason why it works is unknown.

Cohort study

Does smoking increase your risk of getting lung cancer in 10 years?

Researchers who want to investigate this question face a hurdle right away: a randomized controlled trial is impossible. This is because it is logistically difficult, not to mention unethical, to assign people into a group asked to smoke for the next 10 years, and another asked to abstain from smoking for the same duration. To answer this question, you would need to conduct your research using an approach called a *cohort study*.

How to perform a cohort study

First, you would need to gather a large sample of people who smoke. Then, you would need to gather an equally large number of non-smokers, who should be as similar as possible to the smokers in terms of their background characteristics (age, sex, etc.). Ten years later, you would count the number of people in each group who got lung cancer during that timeframe. Let’s say you examined 100 smokers, of whom 20 developed lung cancer during the 10-year observation period (20%), and 100 non-smokers, of whom 10 did (10%). We can see that smokers have twice the probability, or *risk*, of developing lung cancer when compared with non-smokers.

However, the risk of lung cancer could have been influenced by other factors other than smoking. For example, maybe the smokers were older than the non-smokers. This might mean that the increased risk of lung cancer in the first group wasn’t due to smoking, but rather age. Therefore cohort studies should compare people who are as similar as possible (in terms of age, sex, etc.) among the treatment groups.

In such situations, you can run multivariate analyses to remove the effects of age to a certain degree, and indeed many medical studies do this.

Note: The above value is not the actual risk for lung cancer; it is for illustrative purposes only.

Case report

Doctors might write a *case report* when just one patient gets better from a new treatment.

Case reports don't just describe the patient and how he or she was treated. They also describe their condition before and after treatment in detail, and report the specific elements of it while ensuring the information contains no errors.

Significance of case reports

Such case reports are published at medical conferences and as scholarly papers, to attract the attention to other doctors and researchers. This allows the authors to obtain valuable critical opinions of their research, and reveals aspects of the report that need to be supplemented or revised. The information provided by case reports can serve as a valuable stimulus for doctors, researchers, and large hospitals, prompting them to conduct randomized controlled trials and observational studies to examine the condition or treatment in question, and determine whether or not the research is scientifically accurate.

2.6. Study Questions

Please select the **incorrect** reason, from the following, why testimonials are an unreliable source of health information. (*Choices are displayed in random order)

- Testimonials alone are not trustworthy: website owners publish only those that claim a product or treatment has positive health effects, and not those that report no benefit.
  - Commentary: Testimonials are unreliable because site owners only publish the ones that suit their own purposes.
- Testimonials are unreliable because they can be based on author misconceptions.
  - Testimonials can contain errors: for example, a user mistaking the name of their disease.
- Gathering a large number of testimonials can yield real medical knowledge, but cannot yield significant conclusions if there are too few.
  - [Correct answer] Commentary: No matter how many testimonials you gather, that doesn't equal medical knowledge.
- It is difficult to make a judgement about the therapeutic efficacy of a given health tip based on testimonials alone.
  - Commentary: Testimonials alone cannot prove cause-effect relationships.

3. Words of Caution about Sharing Information on Social Media

3.1. Introduction

It has become very easy to share personal information over the Internet. Social media, also known as social networking services (SNS), allow users not only to post their own information, but also share existing information, with just one click. However, that simplicity has a dark side: the increased risk of being a distributor of false information. Social media also makes it easy for people to connect. Increased connections enrich our lives, but also can encourage our beliefs and ideas to harden. This module introduces some harmful effects of social media, and some points to remember when using SNS.

3.2. Exercise Caution When Sharing Information

Don't be too quick to share and spread information

You can share and spread information on social media with the click of a button. This can lead you to share and spread health information that, at the time, you intuitively feel is good or right, but upon closer inspection is actually false. You run the risk of serving as a source of falsehoods when you share and spread health or medicine-related information via social media. Moreover, information travels fast on SNSs, and it's virtually impossible to redact and correct your posts once the false information has spread. Therefore, you should try and observe two guidelines when dealing with health-related information on social media.

1. Don't share or spread health information if it lacks scientific evidence.
2. If you do share or spread health information, include the scientific evidence as well.

3.3. Echo Chambers

What is an "echo chamber"?

Small groups can form in SNSs when similar people connect with one other. When people in those groups receive their information only from other members, it self-reinforces, biasing their opinions and facts and pulling the group consensus in an extreme direction. This phenomenon is called an *echo chamber*. Social media users need to be conscious of the possibility that the SNS groups they frequent may become echo chambers.

Echo chamber formation process

Step 1. Create a group of similar people

SNSs are replete with functions that help their users interact with like-minded people, rather than people with different backgrounds, interests, and opinions. This holds true not just for you, but also

for the people with whom you connect. This tendency results in the formation of groups whose members have largely the same characteristics.

Step 2. Ideas and opinions become biased

Diversity in opinions is lost from such groups because they contain only members with largely the same characteristics. Like-minded people aren't likely to put the brakes on opinions similar to their own, even when the group consensus heads in an extreme direction.

Step 3. People stop questioning the accuracy of information

Since you only hear the opinions of your group, you start to think of them as normal and standard. Gradually, your likelihood to believe a given claim becomes less dependent on the accuracy of the information itself, but rather whether or not it aligns with the opinions circulating in your group.

The harmful effects of echo chambers

Let's consider the hypothetical scenario in which you shared health-related or medical information in an echo chamber. Other members of your group will share and spread your claims, without questioning their accuracy, and the false information will gradually become entrenched. Members might adopt or avoid certain health behaviors, changes that could be harmful, based on that false information. To prevent getting trapped in an echo chamber, it is important that you aim to consume a balanced social media diet, and follow a wide variety of other users to avoid your own opinions and beliefs becoming biased.

3.4. Study Questions

Please select **all of the choices** in the following that demonstrate **incorrect** assumptions about sending and sharing information on social media. (*Choices are displayed in random order)

- False information might spread over an SNS, but later corrections will spread about as widely.
    - [Correct answer] Commentary: Social media users need to beware of spreading false information, because any corrections to the original claims will not spread as far.
- Connecting people is the most important part of social media, so there's no need to be exacting about the accuracy of information you encounter there.
    - [Correct answer] Commentary: Social media users need to ensure they don't re-post or spread information that's been determined to be inaccurate, because the contagiousness of such false claims is only enhanced by connections between people.
- I thought about sharing a health-related article going around on social media, but I decided not to because I couldn't tell whether its claims were right or not.
    - Commentary: It's better not to share or spread information if you can't tell whether it's

accurate.

- I try to have connections with a variety of people on social media so that I can gather information and opinions from many different perspectives.
    - Commentary: Having connections with a variety of people helps to minimize the dangers of echo chambers.

4. Cognitive Biases and Points to Remember When Dealing with Health Information

4.1. Introduction

Viewed objectively, there are times when people believe health claims, even though they shouldn't, based on the poor evidence supporting them. The human brain is known to be vulnerable to certain preconceptions, illusions, or illogical thinking patterns, often called *cognitive biases*, when processing information or acquiring new knowledge. Cognitive bias can make you believe certain health claims despite the supporting information being poor in quality. Cognitive bias can happen to anyone. You must be vigilant, and remember that you too can be affected by such illogical thought processes. This section introduces five such cognitive biases.

4.2. Five Kinds of Cognitive Biases

1. Seeing patterns where there are none

The human brain is skilled at detecting patterns. Take the repeating sequence OXOX, for example. Based on the alternating Os and Xs, you automatically expect the next symbol to be O. This ability is a useful one, but can also be lead to mistaken judgments.

2. Seeing causal relationships where there are none

For example, let's say you took a medicine, then got better afterwards. Based on these facts, you might deduce a cause-effect relationship: i.e., that your health improved thanks to the medicine. However, perhaps you would have gotten better even if you hadn't taken it. The two events—"taking medicine" and "getting better"—simply happened one after the other. The human brain has a tendency to attribute "before-after" associations to "cause and effect." However, we can't know for certain whether the first caused the second unless we research the question using a design that is able to properly validate the causal relationship.

3. Focusing on information that fits the hypothesis

Given the hypothesis, "Is Supplement X effective at treating Disease Y?" people have a tendency to preferentially search for and focus on information that supports the claim. As a result, they tend to overlook or ignore information or statistics that show that Supplement X doesn't really work.

4. Being influenced by preconceptions

Whether or not people trust objective research data is strongly dependent on their original preconceptions. Plenty of reliable research data have been published supporting the therapeutic efficacy of anti-tumor drugs. However, when a person who believes that anti-tumor drugs are ineffective is exposed to data that doesn't match their original beliefs, they will tend to focus on tiny

discrepancies and find fault with the data, and deem the conclusions unreliable.

5. Focusing on easy-to-remember information

Your brain will readily absorb easy-to-remember information, but not information that's quite difficult to recall to begin with. Health information provided by high-quality sources such as universities and the Ministry of Health, Labour and Welfare doesn't come in a format that's easy to remember. However, the writers of unreliable health-related articles have several tricks to make you remember their claims, such as cutting out information to simplify the content, or presenting them in a format that's easy to read. This paradoxical tendency of poor-quality information being easier to remember leads people to focus on the wrong things.

4.3. Points to Remember When Dealing with Health Information

Low-quality, false, and misleading health information is widespread on the Internet. Moreover, human thinking is prone to cognitive biases, which impact our ability to make accurate decisions. Therefore, you must be vigilant when trying to scrutinize and correctly utilize health information.

1. Doubt by default

New treatment methods are not discovered that quickly. You need to be skeptical of articles and websites that publish claims that are excessively revolutionary.

2. Be wary of typical language used by inaccurate claims and low-quality websites

Websites that promote low-quality information often use a certain type of language. Beware of articles that over-use words and language like these:

1. **Oversimplified language**: e.g., "Eating X makes you healthy!"
   Health-related knowledge is almost never determined in such simplistic terms.
2. **Extreme adjectives**: e.g., "Dangerous!", "Amazing!"
   Keywords like these could just be used to earn page views.
3. **Non-technical health terms**: e.g., "Natural," "Traditional," "Makeover," "Immune Power"
   Non-technical terms like these regard concepts or ideas that carry little weight in medical science. Such expressions are easy to remember, and intuitively interesting to the human brain, which results in an unfortunate tendency to regard information containing them as important.

3. Be wary of the identity of the website owner

You need to be vigilant about whether the websites you view have been created by responsible professionals. Websites that lack a description of who owns or operates them could just be money-making ventures.

4. Make sure you're not reading marketing for fake health products

Perhaps the health or medical information you're reading is just an advertisement for a fraudulent medicine or treatment. The real motivation for the website in question might really be to sell you expensive products, despite a lack of health benefits. You should be skeptical of information that overly recommends specific products or clinics. Marketing copy for fraudulent health products or treatments almost never accompanies descriptions of publications to back up their claims with scientific evidence.

5. Regard claims only supported by testimonials as lacking scientific value

Testimonials, no matter how many you find, lack scientific value.

6. Check for published scientific research regarding health claims

Scientific research is published in the form of conference presentations and scholarly papers. Maybe the reason you can't find a publication supporting a health claim is that it lacks a scientific basis.

7. Investigate the content of other websites as well

You should look across several websites when evaluating the reliability of health information you find. You can make better decisions about its trustworthiness by scrutinizing it and comparing the findings reported by different authors.

8. Remember your own vulnerability to cognitive biases

As mentioned in the previous section, everyone can fall into the trap of cognitive bias. You should approach health information not with the conviction that you would never be deceived, but instead with self-awareness and doubt. Always ask yourself, "Is there any chance I could be operating based on a cognitive bias?"

4.4. Study Questions

Please select from the following **all of the choices** that demonstrate **incorrect** assumptions about cognitive biases. (*Choices are displayed in random order)

- The human brain is excellent at discovering patterns and causal relationships.
  - Commentary: The human brain is indeed adept at these skills. However, they have negative consequences as well, making us prone to cognitive biases.
- When you read through health information, you should try to interpret the claims based on a preconceived hypothesis.
  - [Correct answer] Commentary: This approach will lead you to unconsciously pick and choose health information that fits your preconceived hypothesis. You need to set aside your

preconceptions while you consider the information presented.

- People tend to focus on information that's easy to remember. With this in mind, I tried to take notes on all of the health information I read during my research, so I would be sure to remember even the unintuitive parts later.
    - Commentary: People tend to make final decisions based purely on easy-to-remember information. Taking notes is an effective counter-measure against this bias.
- Since the health information I found in my online search matched my initial thoughts, I quit searching on the assumption that my beliefs were correct.
    - [Correct answer] Commentary: People tend to focus on information that matches their preconceptions. However, you need to consider all of the health information surrounding the topic in question, meaning you should also collect information that does not align with the beliefs you already hold.

4. Summary

1. *Don't latch onto a single site or piece of information.*
   Most health claims have multiple perspectives and interpretations.
2. *Check who wrote the information you're reading.*
   Be particularly skeptical of information when you can't identify the author.
3. *Remember these points when dealing with health information*

Book Guide (Japanese Books)

- サイモン・シン，エツァート・エルンスト（青木薫訳）．代替医療解剖．新潮社．2013. ISBN978-4-10-215976-7
- 中川淳一郎．ネットは基本、クソメディア．株式会社 KADOKAWA．東京 ．2017. ISBN978-4-04-082157-3
- NATROM．「ニセ医学」に騙されないために．メタモル出版．東京．2014.　ISBN978-4-89595-864-6
- ベン・ゴールドエイカー（梶山あゆみ訳）．デタラメ健康科学．株式会社河出書房新社．東京．2011. ISBN978-4-309-25250-6
- 福田洋ら．ヘルスリテラシー :健康教育の新しいキーワード．大修館書店．東京．2016. ISBN978-4469267952.
- 松本和紀．メディア・バイアス．光文社．東京．2007. ISBN978-4-334-03398-9

Book Guide (English Books)

- Simon Singh & Edzard Ernst. Trick or Treatment? Corgi. 2009. ISBN 978-0552157629
- Ben Goldacre. Bad Science. Fourth Estate. 2009. ISBN 978-0007284870
